# Visceral Leishmaniasis IgG1 Rapid Monitoring of Cure vs. Relapse, and Potential for Diagnosis of Post Kala-Azar Dermal Leishmaniasis

**DOI:** 10.3389/fcimb.2018.00427

**Published:** 2018-12-13

**Authors:** Tegwen Marlais, Tapan Bhattacharyya, Om Prakash Singh, Pascal Mertens, Quentin Gilleman, Caroline Thunissen, Bruno C. Bremer Hinckel, Callum Pearson, Bathsheba L. Gardner, Stephanie Airs, Marianne de la Roche, Kiera Hayes, Hannah Hafezi, Andrew K. Falconar, Osama Eisa, Alfarazdeg Saad, Basudha Khanal, Narayan Raj Bhattarai, Suman Rijal, Marleen Boelaert, Sayda El-Safi, Shyam Sundar, Michael A. Miles

**Affiliations:** ^1^Faculty of Infectious and Tropical Diseases, London School of Hygiene and Tropical Medicine London, United Kingdom; ^2^Department of Medicine, Institute of Medical Sciences, Banaras Hindu University Varanasi, India; ^3^Coris BioConcept Gembloux, Belgium; ^4^Department of Biomedical Sciences, University of Antwerp Antwerp, Belgium; ^5^Departamento de Medicina, Universidad del Norte Barranquilla, Colombia; ^6^Faculty of Medicine, University of Khartoum Khartoum, Sudan; ^7^Department of Microbiology, B.P. Koirala Institute of Health Sciences Dharan, Nepal; ^8^Department of Internal Medicine, B.P. Koirala Institute of Health Sciences Dharan, Nepal; ^9^Department of Public Health, Institute of Tropical Medicine Antwerp, Belgium

**Keywords:** visceral leishmaniasis, serology, treatment, relapse, cure, IgG1, RDT, PKDL

## Abstract

**Background:** There is a recognized need for an improved diagnostic test to assess post-chemotherapeutic treatment outcome in visceral leishmaniasis (VL) and to diagnose post kala-azar dermal leishmaniasis (PKDL). We previously demonstrated by ELISA and a prototype novel rapid diagnostic test (RDT), that high anti-*Leishmania* IgG1 is associated with post-treatment relapse versus cure in VL.

**Methodology:** Here, we further evaluate this novel, low-cost RDT, named VL Sero K-SeT, and ELISA for monitoring IgG1 levels in VL patients after treatment. IgG1 levels against *L. donovani* lysate were determined. We applied these assays to Indian sera from cured VL at 6 months post treatment as well as to relapse and PKDL patients. Sudanese sera from pre- and post-treatment and relapse were also tested.

**Results:** Of 104 paired Indian sera taken before and after treatment for VL, when deemed clinically cured, 81 (77.9%) were positive by VL Sero K-SeT before treatment; by 6 months, 68 of these 81 (84.0%) had a negative or reduced RDT test line intensity. ELISAs differed in positivity rate between pre- and post-treatment (*p* = 0.0162). Twenty eight of 33 (84.8%) Indian samples taken at diagnosis of relapse were RDT positive. A comparison of Indian VL Sero K-SeT data from patients deemed cured and relapsed confirmed that there was a significant difference (*p* < 0.0001) in positivity rate for the two groups using this RDT. Ten of 17 (58.8%) Sudanese sera went from positive to negative or decreased VL Sero K-SeT at the end of 11–30 days of treatment. Forty nine of 63 (77.8%) PKDL samples from India were positive by VL Sero K-SeT.

**Conclusion:** We have further shown the relevance of IgG1 in determining clinical status in VL patients. A positive VL Sero K-SeT may also be helpful in supporting diagnosis of PKDL. With further refinement, such as the use of specific antigens, the VL Sero K-SeT and/or IgG1 ELISA may be adjuncts to current VL control programmes.

## Introduction

Visceral leishmaniasis (VL; kala-azar), is caused by the protozoan parasites *Leishmania donovani* in Asia, Africa and the Middle East and *Leishmania infantum* in Europe and South America. These parasites are transmitted by blood-feeding female phlebotomine sand flies. Symptomatic VL is usually fatal if untreated. Symptoms include prolonged fever >14 days, wasting, splenomegaly, hepatomegaly and anemia (Sundar and Rai, 2002). While VL is present in about 75 countries, the majority (90%) of cases in 2015 occurred in India, Sudan, South Sudan, Ethiopia, Somalia, Kenya, and Brazil (World Health Organization, 2017), where it is closely linked to poverty, both as cause and effect (Boelaert et al., 2009; Sarnoff et al., 2010).

Following clinical suspicion of VL, serology is used for diagnosis. Techniques vary by region and include the immunofluorescence antibody test (IFAT), direct agglutination test (DAT), enzyme linked immunosorbent assay (ELISA), and detection of IgG against recombinant antigens rK39 or rK28 (Singh and Sundar, 2015). In India the DAT and rK39 serology are used, with a positive result in either test indicative of exposure to infection with *L. donovani*. For confirmatory parasitological diagnosis, seropositive individuals undergo spleen, bone marrow or lymph node biopsy to search for the intracellular amastigote stage in films of Giemsa-stained aspirates. These are invasive, costly and potentially hazardous techniques with low and variable sensitivities ranging from 53 to 99% (Singh and Sundar, 2015).

VL is treated with antimonials, miltefosine, paromomycin, amphotericin B, liposomal amphotericin (AmBisome) or drug combinations (World Health Organization, 2010). Currently, post-treatment outcome is determined by assessment of clinical signs and symptoms, initially on the last day of drug treatment and, in India, again 6 months after administration of the last dose (World Health Organization, 2010). Possible outcomes are: cure; relapse; death (by VL or not); post kala-azar dermal leishmaniasis (PKDL); loss to follow up. However, recent studies from India and Nepal have reported relapse rates of between 1.4 and 20%, including up to and beyond 12 months following the end of treatment (Burza et al., 2013, 2014; Rijal et al., 2013). In Sudan, relapse rates around 6% have been reported (Gorski et al., 2010; Atia et al., 2015). Patients who relapse face a further biopsy procedure to confirm the presence of parasites.

PKDL is a non-painful sequela of VL occurring in over 50% of cases in Sudan (Zijlstra et al., 2003) but is far less prevalent in South Asia (Zijlstra et al., 2003; Uranw et al., 2011). PKDL is less reported in *L. infantum* endemic regions where cases have mostly been associated with HIV/AIDS (Ridolfo et al., 2000; Bittencourt et al., 2003; Celesia et al., 2014), other co-infections (Trindade et al., 2015) or immune suppression (Roustan et al., 1998). PKDL manifests between 0.5 months to one or more years after apparently successful VL treatment (Musa et al., 2002; Uranw et al., 2011; Singh et al., 2012; Moulik et al., 2017) and may occasionally occur without a prior episode of VL (el Hassan et al., 1992; Zijlstra et al., 2003; Das et al., 2012, 2016). PKDL is suspected based on dermal manifestations that are non-specific and diagnosis is made on previous VL treatment history and confirmed parasitologically by microscopy of slit skin smear or biopsy or PCR (Zijlstra et al., 2017). Conventional serology is likely to remain positive from the earlier VL and there is no test in use to predict PKDL (Gidwani et al., 2011). The high parasite density in PKDL skin provides a source of infection to sand flies and thus sustains long term transmission and endemicity (Molina et al., 2017; Mondal et al., 2018).

An unresolved crucial question is how to identify asymptomatic infected individuals simply and reliably (as defined by seropositivity, lack of clinical symptoms and no prior history of VL) who will progress to active VL. High DAT and/or rK39 ELISA titres have been associated with increased risk of progression in the Indian subcontinent but as yet there is no single rapid test in use for this purpose (Hasker et al., 2014; Chapman et al., 2015). To improve outcome monitoring of VL and disease control, the World Health Organization has identified the vital need for a marker of post-chemotherapeutic cure, and the high priority incorporation of such a biomarker into a point-of-care rapid diagnostic test (RDT) (World Health Organization, 2012). Such a test should meet the “ASSURED” criteria of being: affordable; sensitive (few false negatives); specific (few false positives); user-friendly, requiring minimal training; rapid; robust, not requiring cold-storage; equipment-free, and deliverable to those who need it (Peeling et al., 2006).

We have previously shown that high anti-*Leishmania* IgG1 ELISA titers are associated with treatment failure, whereas, in cases deemed to be cured following chemotherapy, IgG1 levels diminish significantly by 6 months post-treatment and only IgG1 gave this level of discrimination (Bhattacharyya et al., 2014a). We demonstrated this by ELISA against *L. donovani* whole cell lysate, and then adapted the assay to a prototype lateral flow immunochromatographic RDT. Here, we present further evaluation of this RDT, called VL Sero K-SeT, to indicate cure after VL treatment in a larger, paired, sample set and to confirm relapse. We also performed western blot on the same sample set. Additionally we show the potential utility of VL Sero K-SeT and other IgG1 assays to confirm PKDL.

## Methods

### Ethics Statement

In India, the collection of samples was approved by the Ethics Committee of Banaras Hindu University, Varanasi. In Sudan approval was by the Ethical Research Committee, Faculty of Medicine, University of Khartoum and the National Health Research Ethics Committee, Federal Ministry of Health, Sudan. Written informed consent was obtained from adult subjects included in the study or from the parents or guardians of individuals <18 years of age. In Nepal, informed consent was obtained from all the participants and the ethical committee of the B.P. Koirala Institute of Health Sciences (BPKIHS) approved the study. This research was also approved, as part of the EC NIDIAG project, by the London School of Hygiene and Tropical Medicine Ethics Committee.

### Sources of Sera/Plasma

We retrospectively selected sera or plasma from an archive of different VL disease states. Samples had been collected in VL endemic regions, namely Muzaffarpur in Bihar, India after 2007 and in 2013 in Gedaref, Sudan. Sample sizes used during this evaluation were dependent on availability of appropriate samples and reagents.

In India, cases of VL had been diagnosed by positive rK39 serology and/or parasitologically by microscopy of splenic aspirates. In Sudan active cases of VL had been diagnosed by microscopy of bone marrow or lymph node aspirates in conjunction with serological assays. These diagnoses were made according to their respective national procedures, prior to the present study. Sera/plasma were stored at −80°C until use. All patients were HIV negative. We have previously observed that serum and plasma derived from the same sample show no difference in IgG titer in ELISA against *L. donovani* lysate (unpublished observations), although we have not specifically assessed IgG1 with both sample types.

### India

Indian sample types are described in Table 1. We have previously found that in Indian VL, IgG1 titer up to day 30 post-treatment initiation is not statistically significantly different from pre-treatment (Bhattacharyya et al., 2014a) and therefore we consider these as “pre-treatment” in paired samples for the purposes of this study. Treatment of VL was with single-dose AmBisome alone or with 10 days of miltefosine. PKDL was treated with miltefosine for 84 days. DAT and rK39 ELISA were conducted prior to the present study as part of standard diagnostic procedures in India.

**Table 1:** Indian sample types and total numbers tested by IgG1 assays.

**Sample type**	**Definition**	***n***
Pre- and post-treatment pairs, deemed cured	Treated for VL, with improvement in clinical symptoms and no evidence of relapse at any time 6 months after treatment. Samples were taken at or around the start of treatment and at 6 months.	105 pairs
Relapse	VL treated and subsequently relapsed to active disease. Sampled at the time of relapse diagnosis.	33
PKDL	Samples taken at or up to 30 days after diagnosis of PKDL. Parasite infection was confirmed by PCR or a slit-skin smear or biopsy.	63
Asymptomatic	Asymptomatic seropositive, on the basis of DAT and/or rK39 ELISA, without symptoms or history of VL. Progressors (*n* = 4) developed VL after sampling. Non-progressors (*n* = 4) did not develop VL during follow-up of at least 3 years.	8
Other diseases	Malaria (*n* = 5); tuberculosis (*n* = 5), rheumatoid arthritis (*n* = 1); dengue (*n* = 1); multiple myeloma (*n* = 1).	13
Endemic healthy control	Resident in VL endemic area, seronegative by DAT and rK39 ELISA, no history of VL, healthy.	30

### Sudan

Sudanese paired serum samples (*n* = 17 pairs) were taken on day of diagnosis of VL and at the end of treatment at 11 days (AmBisome), 17 days (sodium stibogluconate (SSG) + paromomycin), or 30 days (SSG only). These samples were previously tested for IgG1 by ELISA (Bhattacharyya et al., 2014a). Additional Sudanese serum samples used in the present study were unpaired treated individuals (*n* = 2) taken an unknown time after treatment, and relapse (*n* = 1). Sudanese Endemic Healthy Control (EHC) samples had previously been tested by the IgG1 ELISA using the same antigen and were negative (Bhattacharyya et al., 2014a) but were not retested here.

### Antigen Production

Whole cell lysate of *L. donovani* strain MHOM/IN/80/DD8 isolated from India, and MHOM/SD/97/LEM3458 isolated from Sudan, was prepared as described previously (Bhattacharyya et al., 2014a). Lysate antigen was used for VL Sero K-SeT development (strain LEM3458), ELISA and western blot (strain DD8). Antigen preparation for western blot strips contained 50 μl of protease inhibitor cocktail (P8340, Sigma, UK) per 1 ml of *L. donovani* cells; centrifugation after sonication was 16,160 × *g* for 45 min at 4°C.

### ELISA for IgG1 Anti *L. donovani*

Duplicate ELISA plates were coated overnight at 4°C with *L. donovani* DD8 strain antigen prepared as above, at 2 μg/ml in coating buffer (35 mM NaHCO_3_, 15 mM NaCO_3_, pH 9.6), 100 μl/well. Plates were washed 3 times with phosphate buffered saline (PBS) + 0.05% Tween 20 (PBST) prior to blocking with 200 μl/well PBS + 2% w/v non-fat milk powder (Premier International Foods, UK) (PBSM) for 2 h at 37°C, followed by three PBST washes. Sera/plasma were diluted 1/100 in PBST+ 2% w/v non-fat milk powder (PBSTM) and applied at 100 μl/well, incubated for 1 h at 37°C then washed 6 times with PBST. Mouse anti human IgG1-horse radish peroxidase (HRP) (ab99774, Abcam, UK) was diluted 1:5,000 in PBSTM and incubated at 100 μl/well, 37°C for 1 h. Plates were washed 6 times with PBST before the addition of 100 μl/well of freshly prepared substrate solution (50 mM citric acid, 50 mM Na_2_HPO_4_, 2 mM *o*-phenylenediamine HCl, 0.009% H_2_O_2_). Plates with substrate were incubated in the dark at room temperature for 10–15 min when the reaction was stopped with 100 μl/well of 1 M H_2_SO_4_ and absorbance read at 490 nm. Each plate contained an EHC sample as a negative serological control for determining the positivity cut-off and a known seropositive VL sample as positive control. All ELISA results reported are the mean A_490_ of duplicate plates.

### RDT Production and Use

Whole cell lysate was prepared as described previously (Bhattacharyya et al., 2014a) from *L. donovani* strain MHOM/SD/97/LEM3458. The VL Sero K-SeT lateral flow immunochromatographic tests were developed at Coris BioConcept and consisted of a cassette with a nitrocellulose membrane, a sample pad, a conjugate pad and an absorbent pad, backed with a plastic strip. The nitrocellulose membranes were sensitized with the *L. donovani* lysate antigen and anti-human IgG1-specific antibody labeled with colloidal gold was dried on the conjugate pad. This strip was housed in a plastic cassette with two windows: the smaller buffer well and the long central test window.

**Figure 1:**
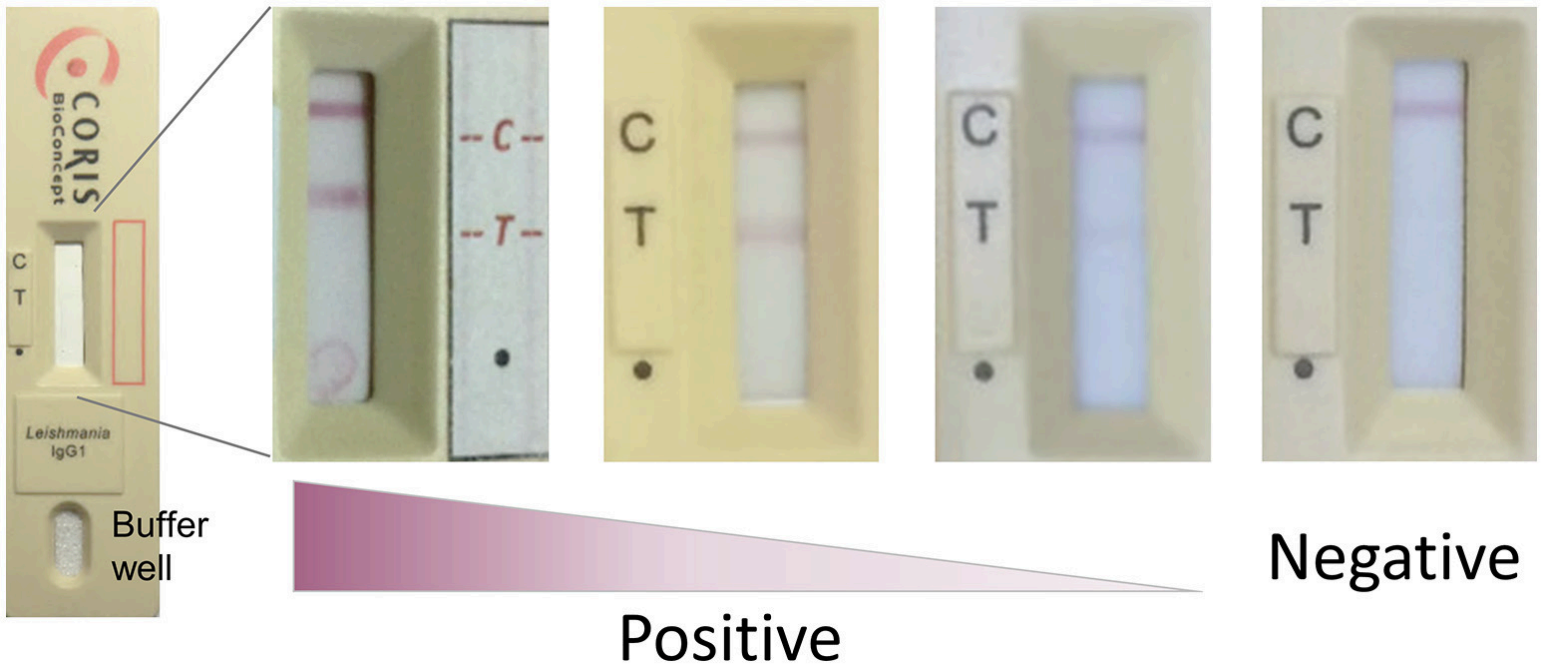
Representative examples of VL Sero K-SeT test line intensity. C, control line; T, test line; dot (∙) indicates where sample is applied. Test strip manufacture was identical despite being housed in different cassettes. Image is best viewed in digital, color format.

To perform the test, 3.5 μl of serum/plasma was applied to the test window at the point indicated by a dot (·) on the cassette, followed immediately by 120 μl of supplied running buffer to the buffer well (Figure 1). Devices were incubated flat, at ambient temperature for 15 min before being assessed visually. Any test line at position T was considered a positive result if a control line was also present at position C. Positive test line intensity was assessed visually for samples from pre- and post-treatment VL (Figure 1). A subset of samples was tested on different batches of the VL Sero K-SeT. Readers of the RDTs were blinded to all the corresponding ELISA results.

### Western Blotting

Western blots were performed to visualize antigen recognition in patients from the different clinical groups, as described in Supplementary Material S1. Briefly, tricine SDS-PAGE gels were made as per Schägger (2006). *L. donovani* DD8 lysate was used as antigen with sera/plasma diluted 1 in 300 (Sudan) or 1 in 400 (India) and detection was by mouse anti human IgG1-HRP.

### Statistical Analysis

We performed a two-tailed Fisher's exact test on Indian VL Sero K-SeT and IgG1 ELISA data to calculate *p*-values between samples from pre- and matched 6 months post-treatment (deemed cured), separately between post-treatment and relapse, and between post-treatment and PKDL. Cut-off for the IgG1 ELISA was calculated as the mean absorbance of the EHC samples plus 3 standard deviations.

## Results

### IgG1 Diminishes by 6 Months in Cured VL Patients

**Figure 2:**
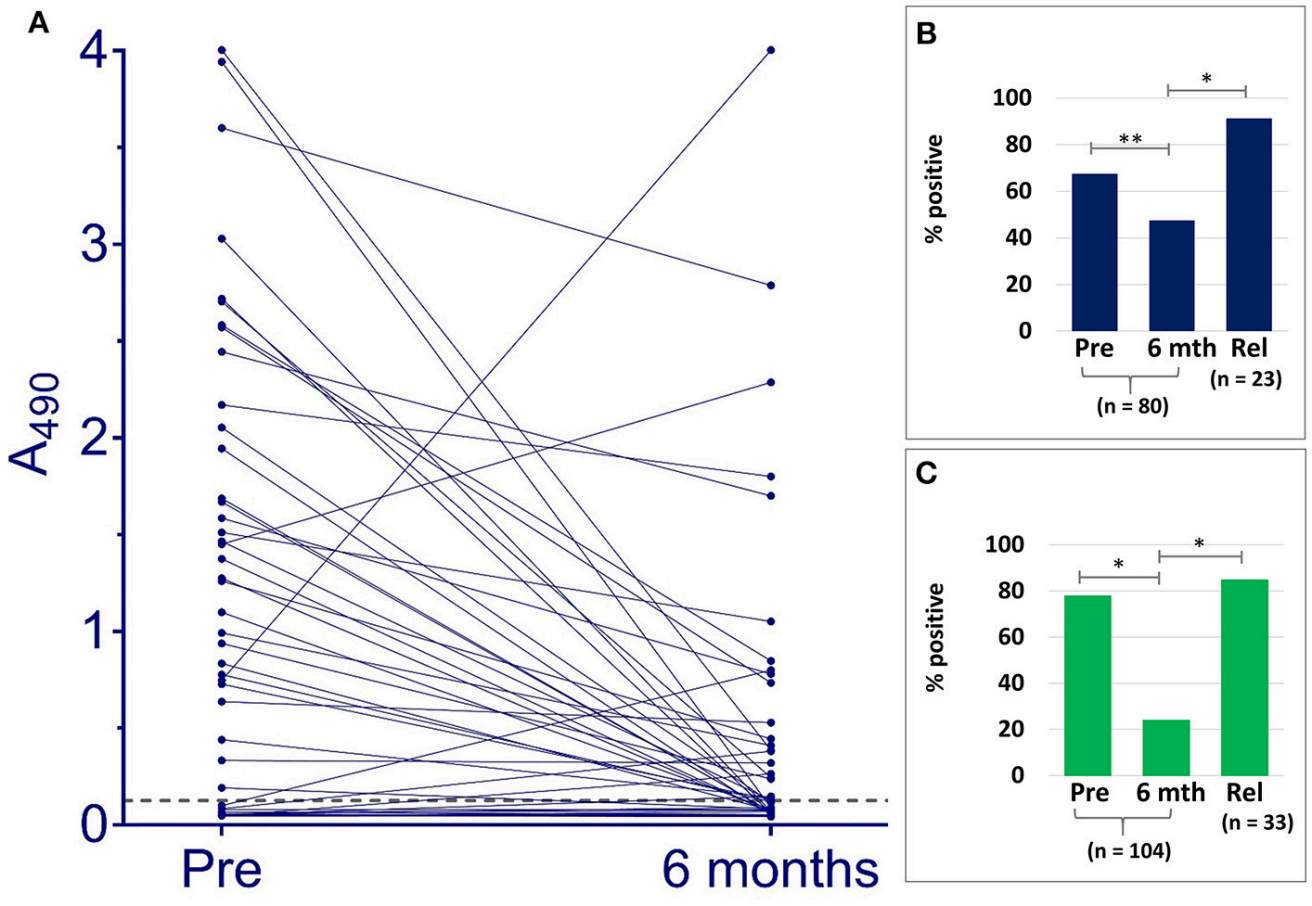
IgG1 anti *L. donovani* assays with Indian VL samples detect differences according to treatment outcome. **(A)** ELISA A_490_ change between paired pre-treatment samples and at 6 months post-treatment when deemed cured. Dashed line indicates cut-off (A_490_ = 0.128). Positivity rates with paired pre-treatment and cured samples at 6 months (6 mth), and non-paired relapse (Rel) for **(B)** ELISA, **(C)** VL Sero K-SeT. ^*^p < 0.0001, ^**^p = 0.0162.

Samples taken from Indian patients before or at the outset of therapy, were compared by VL Sero K-SeT and IgG1 ELISA with paired samples taken 6 months later when the individuals were deemed cured. Both IgG1 assay methods showed a statistically significant difference in positivity rate between pre- and post-treatment samples (*p* = 0.0162 and *p* < 0.0001 for ELISA and RDT, respectively) (Figure 2). A consistent and strongly significant difference was also observed between cured vs. relapsed samples (*p* < 0.0001), again with both IgG1 assay methods (Figure 2).

A subset of pre- and post-treatment of the cured pairs samples (*n* = 87) was tested on different batches of the VL Sero K-SeT, with agreement between individual RDTs of 92.0% (80/87).

### Changes in IgG1 Levels by ELISA and VL Sero K-SeT

ELISA absorbance and corresponding VL Sero K-SeT results for individual samples are given in Supplementary Material S2. Of the 80 Indian paired samples tested for anti *L. donovani* IgG1 by ELISA, 54 (67.5%) were positive before treatment. Of these, 51/54 declined in titer: 21/51 (representing 26.3% of the total 80) went from positive to negative and 30/51 (representing 37.5% of the total 80) had reduced IgG1 at 6 months when deemed cured (Figure 2 and Table 2). Twenty one (26.3%) paired cured sera were negative by IgG1 ELISA before treatment and remained so at 6 months.

**Table 2:** Change in IgG1 response of pre- and post-treatment paired samples from India and Sudan.

**Change in IgG1 response**	**ELISA A_**490**_**	**VL Sero K-SeT test line intensity**	
	**India *n* (%)**	**India *n* (%)**	**Sudan *n* (%)**
Positive to negative	21 (26.3%)	56 (53.8%)	7 (41.2%)
Positive clear decrease	30 (37.5%)	12 (11.5%)	3 (17.6%)
Positive no clear decrease	3 (3.8%)	13 (12.5%)	3 (17.6%)
Negative no change	21 (26.3%)	23 (22.1%)	3 (17.6%)
Negative to positive	5 (6.3%)	0 (0%)	1 (5.9%)
Total	80 (100%)	104 (100%)	17 (100%)

Overall, including those negative at the start, at 6 months after treatment 79/104 (76.0%) were negative by VL Sero K-SeT (Table 2). VL Sero K-SeT results were additionally assessed according to whether the Indian 6 month post-treatment (cure) sample had a decreased or not decreased test line intensity compared to the paired pre-treatment sample. Of the 104 paired samples tested by VL Sero K-SeT from deemed cured Indian VL patients, 81 (77.9%) were positive at start of treatment (Table 2); of these, 68/81 (84.0%) had either become negative or had a visibly reduced test line intensity at 6 months when deemed cured. Thirteen (12.5%) initially RDT positive individuals showed no visible decrease in RDT band intensity at 6 months, despite being deemed cured, and none became positive from negative.

Ninety four percent of samples positive by ELISA at pre-treatment, decreased in seropositivity; for VL Sero K-SeT, this proportion was 84%. However, at 6 months post-treatment, the ELISA was more likely to remain positive than the RDT, using the cut-off value established for the IgG1 ELISA.

Seventy nine Indian samples were tested by both VL Sero K-SeT and ELISA. Of these samples, the RDT was more likely than the ELISA to be positive with pre-treatment samples (78.5 vs. 67.1%) and negative with 6 month samples (78.5 vs. 53.2%). Of samples which remained positive at 6 months by both methods (*n* = 14), the change in intensity of RDT test line generally mirrored the change in ELISA absorbance value for the same sample. Three of the Indian samples increased markedly in IgG1 titer by ELISA at 6 months (Figure 2). Two of these accorded with a corresponding rise in VL Sero K-SeT test line intensity; for the third sample, both pre-treatment and 6 month RDTs were negative (Figure 2A).

Sudanese paired samples taken before and immediately after treatment (11–30 days later) were similarly assessed (Table 2). For Sudanese paired samples prior to treatment, 13/17 (76.5%) were positive by VL Sero K-SeT and at completion of treatment, 10/13 (76.9%) had a negative or reduced test line intensity (Table 2). If taken as a single time point at the end of treatment, 10/17 (58.8%) Sudanese VL patients had negative VL Sero K-SeT result. Four (23.5%) of the Sudanese treated individuals were negative pre-treatment, similar to the proportion of Indian samples (22.1%). Two additional un-paired treated Sudanese samples were negative by RDT (not shown).

### IgG1 Western Blot Confirmed Negative/Declined RDT in Cure

For a subset of 25 of the paired Indian samples, western blots mirrored the VL Sero K-SeT RDT findings, in that IgG1 declined dramatically in all but one VL patient at 6 months follow up after treatment (Supplementary Material S3). As with the RDT, the blots showed that samples that were positive and detecting many antigens before treatment had become negative or reduced in intensity by 6 months. Corresponding RDT images are shown in Supplementary Material S4.

### Elevated IgG1 in VL Relapse

For 33 Indian patients for whom we had unpaired samples at the time of relapse, the VL Sero K-SeT was 84.8% (28/33) positive and ELISA 91.3% (21/23) positive, confirming relapse. Of the 23 samples tested by ELISA that were also tested by RDT, 19 gave the same result by both assays (Supplementary Material S2). The single available Sudanese relapse sample was IgG1 positive (Supplementary Material S5). Twenty five of the Indian samples and the single Sudanese sample were also tested on western blot for IgG1 against *L. donovani* lysate antigen and showed concordance between the RDT and blots (Supplementary Materials S2, S5). For two of the 33 Indian relapse samples, a paired pre-treatment sample was available. Both individuals were VL Sero K-SeT positive at both time points.

All samples from other diseases, namely malaria, tuberculosis, dengue fever, rheumatoid arthritis, and multiple myeloma were negative by VL Sero K-SeT, as were all samples from endemic healthy controls.

### VL Sero K-SeT Can Provide Evidence for PKDL but Not for Its Cure

Of the 63 PKDL samples tested, 49 (77.8%) were positive by VL Sero K-SeT and of the subset of 45 tested by IgG1 ELISA, 43 (95.6%) were positive (Supplementary Material S2). A subset of 10 VL Sero K-SeT-positive PKDL samples were tested by western blot, of which 9 showed discernible bands. Images of the blots and their corresponding VL Sero K-SeT RDTs are shown in Supplementary Material S6. There was a highly statistically significant difference between post-treatment cured samples at 6 months and PKDL by both VL Sero K-SeT and IgG1 ELISA (Fisher's exact *p* < 0.0001 for both assays).

Seventeen of the 63 individuals with PKDL provided between 1 and 5 additional sequential follow-up samples over intervals ranging from 15 to 365 days post-treatment. These PKDL post-treatment sequential samples retained the initial RDT result in 12/17 (70.6%) cases, decreased in 3/17 (17.6%), increased slightly in one case (5.9%) and varied between positive and negative over time in one case (5.9%).

### IgG1 Can Indicate Progression From Asymptomatic Status

When samples from asymptomatic seropositive individuals who later progressed to symptomatic disease (progressors, *n* = 4) were tested on the VL Sero K-SeT, all gave a positive test result (Supplementary Material S7). In contrast, 4 individuals who were seropositive but did not develop symptomatic VL were negative by VL Sero K-SeT. Thus, in our limited sample size, elevated IgG1 levels, as detected by VL Sero K-SeT, were associated with progression to symptomatic disease. This result was corroborated by ELISA and western blot (Supplementary Material S7).

## Discussion

Conventional serology for VL diagnosis relies on detecting the overall IgG response. This has been reported to remain elevated, often for years, after treatment (Bhattarai et al., 2009; Gidwani et al., 2011; Srivastava et al., 2013). This makes current serology unsuitable for timely monitoring of treatment outcome. We have previously found using ELISA that a decreased or negative anti *Leishmania* IgG1 titer at 6 months post-treatment can be indicative of VL cure, whereas elevated IgG1 levels are associated with post-chemotherapeutic relapse (Bhattacharyya et al., 2014a).

### Monitoring of Post-treatment Outcomes

Here we used a larger panel of paired samples to assess the IgG1 response as detected by the rapid test, VL Sero K-SeT, where 77.9% of Indian samples were positive before treatment and of these 69.1% had become negative 6 months later when deemed cured (Table 2). In total, 76% of 6 month samples were negative, a significant difference from pre-treatment (*p* < 0.0001). Of those still positive at 6 months using this RDT, we found that a diminished test line intensity was also consistent with cure. This decline was corroborated by ELISAs, and despite slight differences in the antigen preparations. We have found no difference in performance of the VL Sero K-SeT when DD8 strain antigen is used instead of LEM3458 (unpublished observations). Thus, the VL Sero K-SeT is a promising innovation, although there is a need to improve further its discriminative capacity.

Sudanese samples declined from positive to negative or decreased VL Sero K-SeT test line intensity in 76.9% of patients immediately after treatment, no more than 30 days after the first sample. This apparently rapid drop in IgG1 was not seen in Indian samples and could be due to the overall lower IgG titer observed in Sudanese samples (Bhattacharyya et al., 2014b; Abass et al., 2015). Thus, a small drop in IgG1 titer could have taken these samples below the detection limit of the VL Sero K-SeT. This may suggest that the VL Sero K-SeT can be used before 6 months to indicate cure or relapse in eastern Africa. The unexpectedly low sensitivity of the VL Sero K-SeT at the start of treatment for both Indian (77.9%) and Sudanese (76.5%) samples does not hinder the subsequent assessment of cure at 6 months, because a negative IgG1 result at 6 months can indicate cure. In addition, we do not propose to use IgG1 assays as a diagnostic for active VL but rather to assist with confirming cure, relapse and PKDL, all of which currently lack an appropriate diagnostic test. With Indian samples, where there was discrepancy between VL Sero K-SeT and ELISA, the RDT was generally more accurate, being positive with pre-treatment and negative with 6 month samples (Supplementary Material S2). As for the Indian sera, the strength of RDT test line intensity broadly corresponded with ELISA signal for an individual sample.

Elevated levels of IgG1 were associated with VL relapse in both assays here for Indian samples. Likewise, the single Sudanese relapse patient was positive by VL Sero K-SeT, whilst 2 treated individuals were negative. We do not know the length of time between treatment and relapse for relapsed individuals (India and Sudan), or the outcome of treated Sudanese individuals. Burza et al. (2014) advised that patient follow-up should be extended from 6 to 12 months as 50–85% of relapses have been found to occur 6 to ≥12 months post-treatment (Rijal et al., 2013; Burza et al., 2014). Our evaluations of a limited number Nepalese relapse samples eluted from filter paper indicated that, although encouraging, elution volumes and conditions need further optimisation before they can be more extensively used with VL Sero K-SeT (data not shown).

We found that in Indian cases who relapsed, the RDT positivity rate was significantly different from 6 month samples from patients deemed cured (*p* < 0.0001). Thus, the VL Sero K-SeT, with Indian samples, can contribute to distinguishing patients deemed cured from those who have relapsed. Of the 13 Indian patients deemed cured at 6 months but who had no clear decrease in VL Sero K-SeT test line intensity (Table 2), none is known to have relapsed with VL. However, the quantitative ELISA did detect an IgG1 decrease in these samples, consistent with cure. Apparent relapses might however, occasionally include re-infections given the highly endemic locations (Morales et al., 2002). Although beyond the scope of the present study, the inclusion of parasite genotyping in a future study would be an advantage.

Cases co-infected with HIV and VL were not included in the present study. Serological diagnosis is less reliable in HIV/VL co-infection (Cota et al., 2012; Abass et al., 2015) and the dynamics of IgG1 response in HIV/VL co-infections need to be determined. Other techniques such as a loop mediated isothermal amplification (LAMP) or qPCR detecting parasite DNA might have the potential to discriminate cure from relapse in HIV/VL patients but are currently less accessible than immunological tests (Mukhtar et al., 2018).

### PKDL

Indian individuals with PKDL tested here were defined as being with or without a previous history of VL, presenting with a dermal macular, papular or nodular rash often starting on the face with further spread to other parts of the body without loss of sensation. VL Sero K-SeT and IgG1 ELISA results suggest that these assays might contribute to PKDL case detection, as found by a study by Saha et al. (2005), whereas conventional serology may be of limited utility (Gidwani et al., 2011). Our data did not assess the predictive value of IgG1 for development of PKDL.

Where the information was available with our sample set, we did not observe an association between elevated IgG1 and macular vs. polymorphic PKDL presentation, this is in contrast to the report of Mukhopadhyay et al. (2012). For a subset of these PKDL samples, we also tested sequential samples taken up to 1 year after the initial sample. We did not observe a consistent decrease in IgG1 after PKDL treatment.

### Progression From Asymptomatic to Active VL

Asymptomatic, seropositive cases outnumber active VL cases (Bern et al., 2007; Ostyn et al., 2011; Hasker et al., 2013; Hirve et al., 2016; Saha et al., 2017) but a proportion are at elevated risk of progressing to active VL (Gidwani et al., 2009; Topno et al., 2010; Ostyn et al., 2011). Asymptomatics have been reported to occasionally have detectable parasites by PCR or culture of blood (le Fichoux et al., 1999; Costa et al., 2002; Bhattarai et al., 2009; Srivastava et al., 2013). Therefore, neither standard seropositivity nor parasitaemia are indicators of progression to clinical disease. Gidwani et al. (2009) found that this progression to VL occurred up to 2 years after serological positivity.

Our limited sample size of seropositive asymptomatic individuals were identified during a community serological screening study, before the present study. Those who later progressed to clinical VL were positive by VL Sero K-SeT and ELISA, whilst those who did not progress were negative by both assays. High titres in both DAT and rK39 ELISA have been indicative of progression in larger studies (Ostyn et al., 2011; Hasker et al., 2014). However, this combination of tests requires laboratory facilities, therefore it would be desirable to have an RDT that could predict progression.

Additional validation of the VL Sero K-SeT should compare larger cohorts who do and do not progress to VL.

### Potential Clinical Application of IgG1 Tests

On the basis of the IgG1 responses reported here by VL Sero K-SeT and ELISA, we propose that IgG1 levels may contribute to monitoring the therapeutic outcome of VL, irrespective of whether there is a pre-treatment sample or result. With further development and validation, IgG1 assays, including the VL Sero K-SeT, which can be produced in large-scale at a cost of a few Euros per test, can be used as an adjunct to the clinical assessment of VL status following treatment. A negative, or defined decrease in IgG1 result at 6 months post treatment in India could be supportive of the clinical assessment of cure. Conversely, an un-paired positive or non-decreased paired positive result at 6 months could indicate the need for additional follow-up. In Sudan, the test may be applicable for defining cure before 6 months. A positive IgG1 result in suspected PKDL or relapse could support the presence of leishmaniasis compared to differential diagnoses. Although western blots were supportive of the use of IgG1, we did not specifically assess banding patterns, and do not propose their use in VL diagnosis. However, we are investigating the discriminative diagnostic potential of antigens separated on acrylamide gels.

### Recommendations for Further Validation of IgG1 Assays

We propose that a prospective study, with extended follow-up of a larger cohort of treated VL patients, should be used to validate the use of IgG1 ELISA and the VL Sero K-SeT for confirming cure in all endemic areas and defining the optimal time for testing, which may differ between regions. This longer follow-up would also indicate the potential of elevated IgG1 to predict relapse and PKDL and in turn, link these with different treatment regimens. A more extensive study of PKDL is required to determine the potential role of IgG1 in identifying PKDL as distinct from leprosy and fungal skin diseases (Saha et al., 2005; Mondal and Khan, 2011). In addition, use of the IgG1 assays on a much larger panel of seropositive asymptomatic individuals would help to define its role in predicting progression to VL. In all cases, comparison with existing diagnostics, including definitive parasitological methods, would directly assess the advantage of IgG1 assays.

Technical refinement of the VL Sero K-SeT should consider the use of electronic RDT readers to give an objective assessment of test band intensity. In addition, the identification of specific antigens suitable to replace the use of parasite lysate would obviate issues regarding batch-to-batch variation. These developments could improve precision of IgG1 readings and reproducibility. A comparison of whole blood and serum/plasma is also required for point-of-care use, although a study in Bangladesh on various VL RDTs did find high agreement between the two sample types (Ghosh et al., 2015).

## Conclusion

IgG1 assays, particularly in the VL Sero K-SeT RDT format, may be a useful adjunct in the assessment of VL treatment outcome and diagnosis of PKDL, which have been identified as research priorities for VL (World Health Organization, 2012). With additional refinement and validation, the VL Sero K-SeT and IgG1 ELISA could contribute to life-saving follow-up of treated patients and to control programme monitoring, surveillance, and targeting of strategies for long-term control of VL.

## Author Contributions

MM conceived and designed the study. TM, TB, and MM wrote the manuscript. TM, TB, CP, BG, SA, MdlR, KH, and HH performed the experiments, collected data. TM, TB, CP, SA, MdlR, KH, and HH analyzed data. OS, PM, QG, CT, BH, AF, OE, AS, BK, NB, SR, SE-S, and SS provided materials. AF, MM, OS, and PM provided feedback on final draft. MM, TB, and TM supervised the project. MB and MM obtained funding.

### Conflict of Interest Statement

PM, QG, and CT are employed by Coris BioConcept which developed the VL Sero K-SeT. The remaining authors declare that the research was conducted in the absence of any commercial or financial relationships that could be construed as a potential conflict of interest.

## Supplementary Material

Supplementary Material S1 Detailed SDS-PAGE and western blotting methods.

Supplementary Material S2 Spreadsheet with ELISA, RDT & blot results for Indian cured paired, PKDL and relapse samples.

Supplementary Material S3 Images of western blots for Indian paired cured samples.

Supplementary Material S4 Images of VL Sero K-SeT for Indian paired cured samples, corresponding to those in S3.

Supplementary Material S5 Images of VL Sero K-SeT and western blots for Indian and Sudanese relapsed samples.

Supplementary Material S6 Images of VL Sero K-SeT and western blots for Indian PKDL samples.

Supplementary Material S7 Images of VL Sero K-SeT and western blots for Indian asymptomatic progressors and non-progressors.
