# Supplementary material for: Visceral Leishmaniasis IgG1 Rapid Monitoring of Cure vs. Relapse, and Potential for Diagnosis of Post Kala-Azar Dermal Leishmaniasis
Source: Front Cell Infect Microbiol. 2018 Dec 13;8:427. doi: 10.3389/fcimb.2018.00427 (PMC6300496; doi:10.3389/fcimb.2018.00427)
Supplement: Supplementary Material S1 — Detailed SDS-PAGE and western blotting methods. [file Data_Sheet_1.pdf]

**Supplementary File S1**

# Materials and Methods for gel electrophoresis and western blotting

## Tricine gel and electroblotting for western blot

Tricine SDS-PAGE gels were made as per Schägger (2006) in a 0.75 mm thick, 14 cm long format apparatus (SG-200, CBS Scientific, USA) with final acrylamide concentrations of 4% and 10% in the stacking and resolving gels respectively. Minor adjustments were to use 50% more volume of the polymerising agents in the 4% stacking gel and the omission of glycerol from the resolving gel. *L. donovani* DD8 lysate antigen was incubated 3 parts with 1 part non-reducing sample buffer at 37°C for 25 minutes, and then loaded at 3 µg protein per 1 mm well width. Precision Plus Dual Color molecular weight marker (1610374, Bio-Rad, UK) was run in a central narrow lane of each gel. Gels were run at 30 V for 55 minutes then at 120 V for a further 245 minutes to give a total running time of 5 hours.

Immediately after running the gels, proteins were transferred to Amersham Protran 0.1 µm pore nitrocellulose (10600046, GE Healthcare Life Sciences, UK) by semi-dry electroblotting in a Trans-Blot SD cell (1703940, Bio-Rad, UK). Blotting buffer (Schägger, 2006) was used to soak blotting paper either side of the gel and nitrocellulose. Transfer was at 60 mA for 120 minutes, after which membranes were air-dried.

After wetting in PBS, membranes were blocked in PBS + 3% w/v non-fat milk powder overnight at 4°C followed by three 5 minute washes in PBST and one wash of PBST + 0.02% w/v sodium azide. Membranes were then air-dried. To make narrow strips of membrane, tape was applied to the top edge of the membrane, above the top of the gel area. The tape was marked at 4 mm intervals and sliced using a paper trimmer (Cathedral Products, UK). Strips were numbered sequentially (including the molecular marker) and stored in airtight containers at 4°C until use.

## Western blot immunoassay for IgG1 anti *L. donovani*

Each strip was immunoassayed with a single sample from individuals with different VL disease states from Sudan and India. Strips were placed separately and in random order, in wells of an 8-well immunotray and were pre-wetted with PBS before adding sera/plasma diluted 1/300 (Sudan) or 1/400 (India) in PBST + 3% non-fat milk powder. Positive and negative control blot strips were run with each batch of individual samples and comprised a pool of 5 VL seropositive or 5 seronegative samples respectively. Samples were incubated for 1 hour at room temperature before being washed with PBST six times for 10 minutes each. Mouse anti human IgG1-HRP (ab99774, Abcam, UK) was incubated on the strips at 1/1000 in PBST+ 3% non-fat milk powder for 1 hour and washed six times

as before. Bands were visualised with Sigma*Fast* DAB with metal enhancer (D0426, Sigma-Aldrich, UK) which was optimised with the blot strips to give final conditions of 1 tablet pair dissolved in 15 ml d$H_2O$ immediately prior to use and incubated on the strips for 4 minutes in darkness before being washed thoroughly with water. Strips were air-dried before being photographed. Blots were considered positive if they showed any bands.
