# Supplementary material for: Visceral Leishmaniasis IgG1 Rapid Monitoring of Cure vs. Relapse, and Potential for Diagnosis of Post Kala-Azar Dermal Leishmaniasis
Source: Front Cell Infect Microbiol. 2018 Dec 13;8:427. doi: 10.3389/fcimb.2018.00427 (PMC6300496; doi:10.3389/fcimb.2018.00427)
Supplement: Supplementary Material S3 — Images of western blots for Indian paired cured samples. [file Data_Sheet_3.pdf]

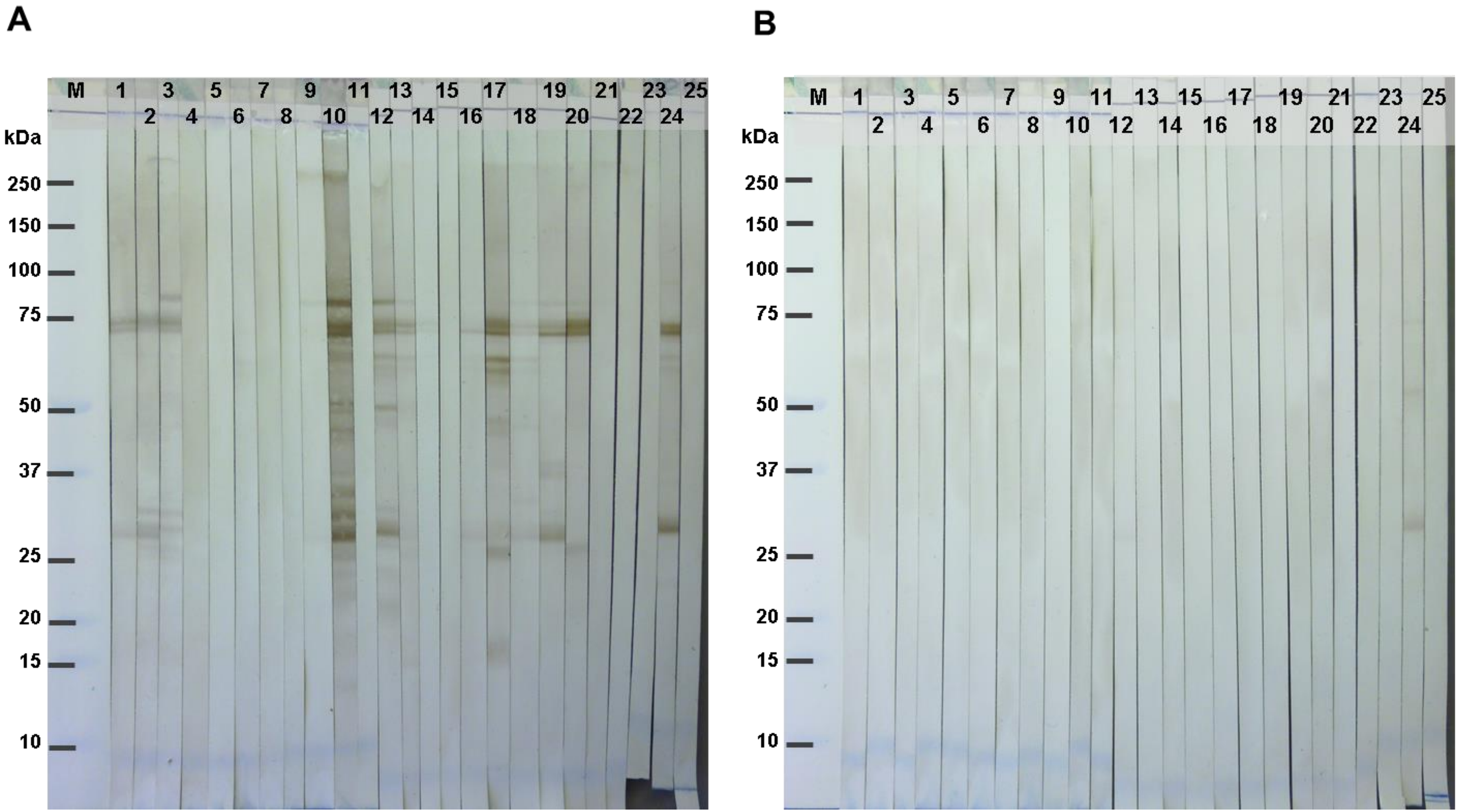

Figure S3. Western blots mirror VL Sero K-SeT results in individuals deemed cured. Western blot showing anti *L. donovani* IgG1 in a subset of 25 paired Indian samples (A) pre-, and (B) 6 months post-treatment. M: Molecular weight marker with kDa marked.
