# Supplementary material for: Visceral Leishmaniasis IgG1 Rapid Monitoring of Cure vs. Relapse, and Potential for Diagnosis of Post Kala-Azar Dermal Leishmaniasis
Source: Front Cell Infect Microbiol. 2018 Dec 13;8:427. doi: 10.3389/fcimb.2018.00427 (PMC6300496; doi:10.3389/fcimb.2018.00427)
Supplement: Supplementary Material S4 — Images of VL Sero K-SeT for Indian paired cured samples, corresponding to those in S3. [file Data_Sheet_4.pdf]

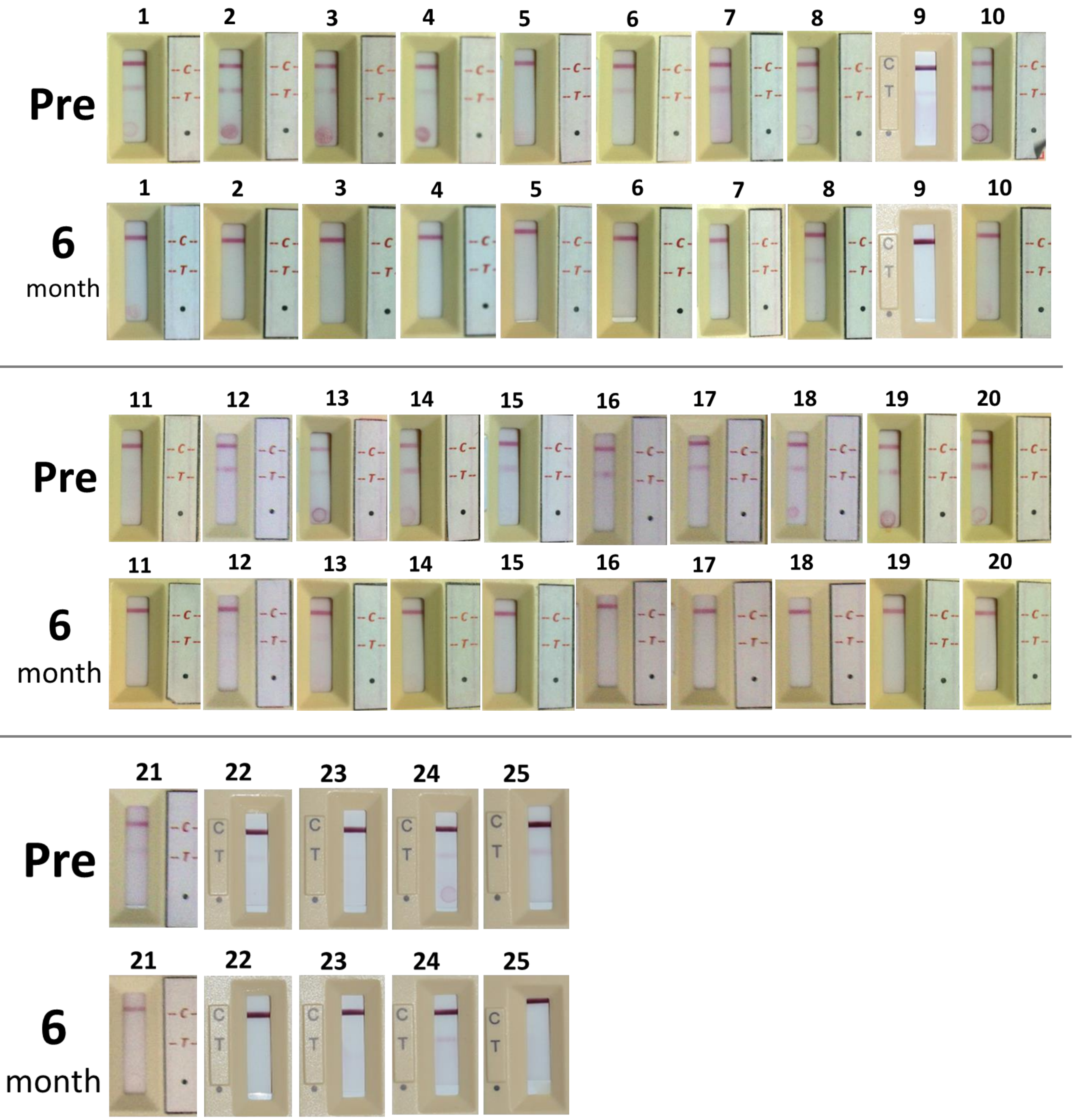

Figure S4. VL Sero K-SeT with Indian paired pre and post treatment samples. Samples were taken at or around start of treatment (pre) and 6 months later when individuals were deemed cured. These samples correspond to those tested by western blot (shown Supplementary Material S3). T: Test line, C: control line.
