# Supplementary material for: Visceral Leishmaniasis IgG1 Rapid Monitoring of Cure vs. Relapse, and Potential for Diagnosis of Post Kala-Azar Dermal Leishmaniasis
Source: Front Cell Infect Microbiol. 2018 Dec 13;8:427. doi: 10.3389/fcimb.2018.00427 (PMC6300496; doi:10.3389/fcimb.2018.00427)
Supplement: Supplementary Material S5 — Images of VL Sero K-SeT and western blots for Indian and Sudanese relapsed samples. [file Data_Sheet_5.pdf]

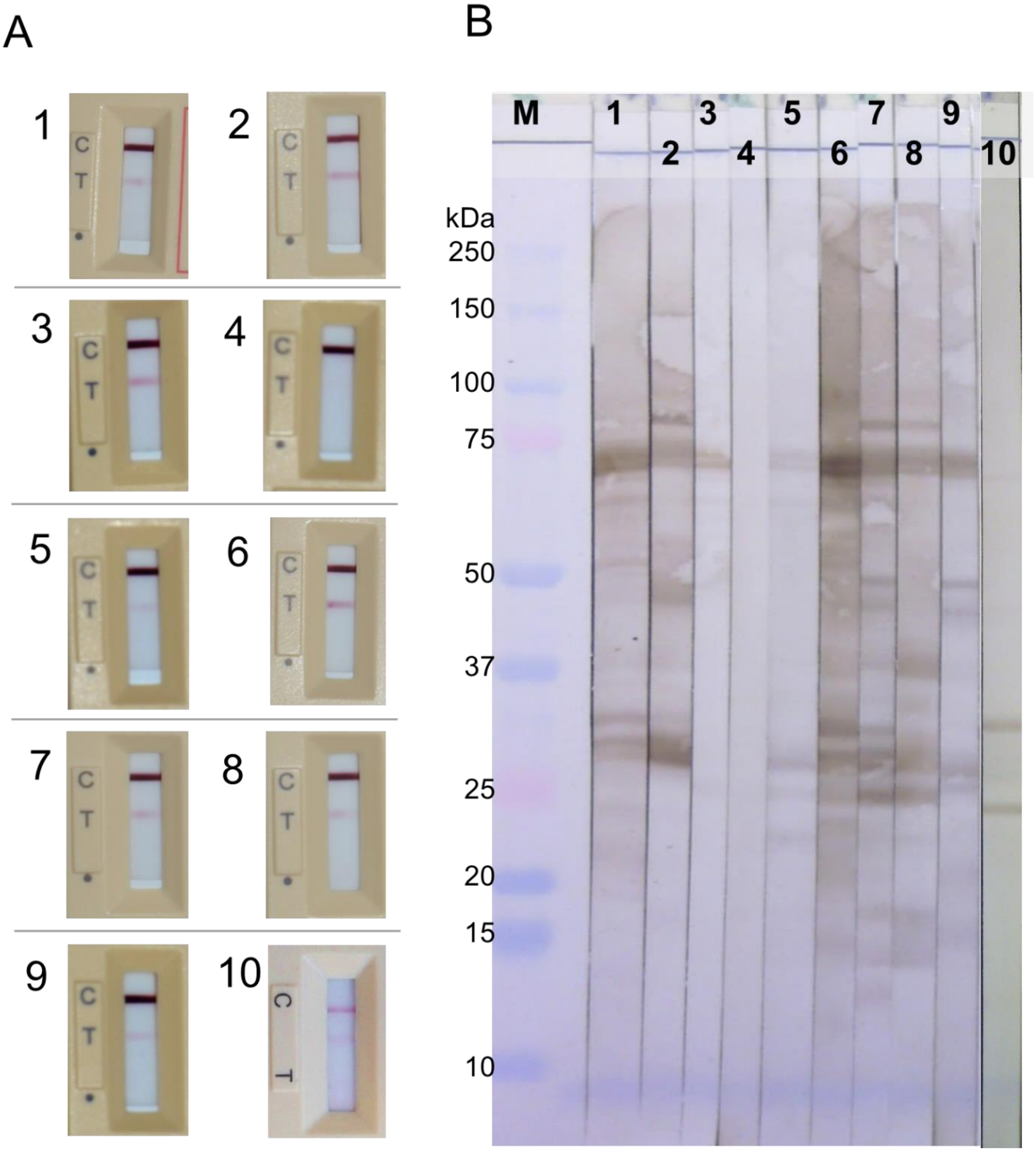

Figure S5. VL Sero K-SeT results for relapsed VL were matched by western blots detecting IgG1 anti *L. donovani*. (A) Representative RDT images (samples 1-9) from a total of 25 Indian relapse samples and the single Sudanese relapse (sample 10) that were tested by VL Sero K-SeT. (B) Western blots of the same samples. C: control line, T: test line, M: Molecular weight marker with kDa given. Sudanese blot number 10 was part of a separate photograph, matched for position using its corresponding molecular marker lane.
