# Supplementary material for: Visceral Leishmaniasis IgG1 Rapid Monitoring of Cure vs. Relapse, and Potential for Diagnosis of Post Kala-Azar Dermal Leishmaniasis
Source: Front Cell Infect Microbiol. 2018 Dec 13;8:427. doi: 10.3389/fcimb.2018.00427 (PMC6300496; doi:10.3389/fcimb.2018.00427)
Supplement: Supplementary Material S6 — Images of VL Sero K-SeT and western blots for Indian PKDL samples. [file Data_Sheet_6.pdf]

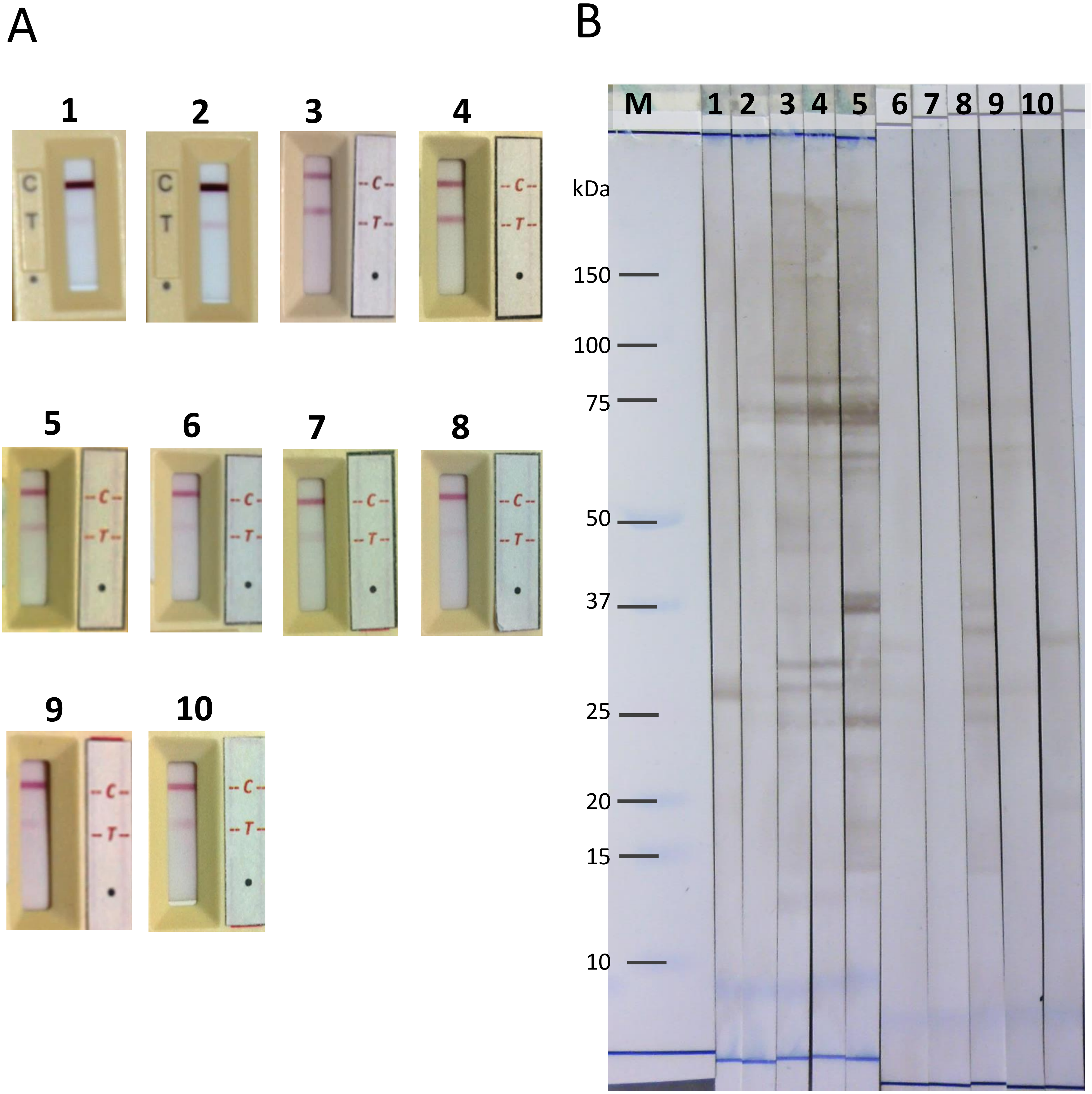

Figure S6. IgG1 anti *L. donovani* in Indian PKDL detected by (A) VL Sero K-SeT and (B) western blot. The same 10 samples were tested by both assays. T: test line, C: control line, M: molecular weight marker with kDa indicated.
