# Supplementary material for: Visceral Leishmaniasis IgG1 Rapid Monitoring of Cure vs. Relapse, and Potential for Diagnosis of Post Kala-Azar Dermal Leishmaniasis
Source: Front Cell Infect Microbiol. 2018 Dec 13;8:427. doi: 10.3389/fcimb.2018.00427 (PMC6300496; doi:10.3389/fcimb.2018.00427)
Supplement: Supplementary Material S7 — Images of VL Sero K-SeT and western blots for Indian asymptomatic progressors and non-progressors. [file Data_Sheet_7.pdf]

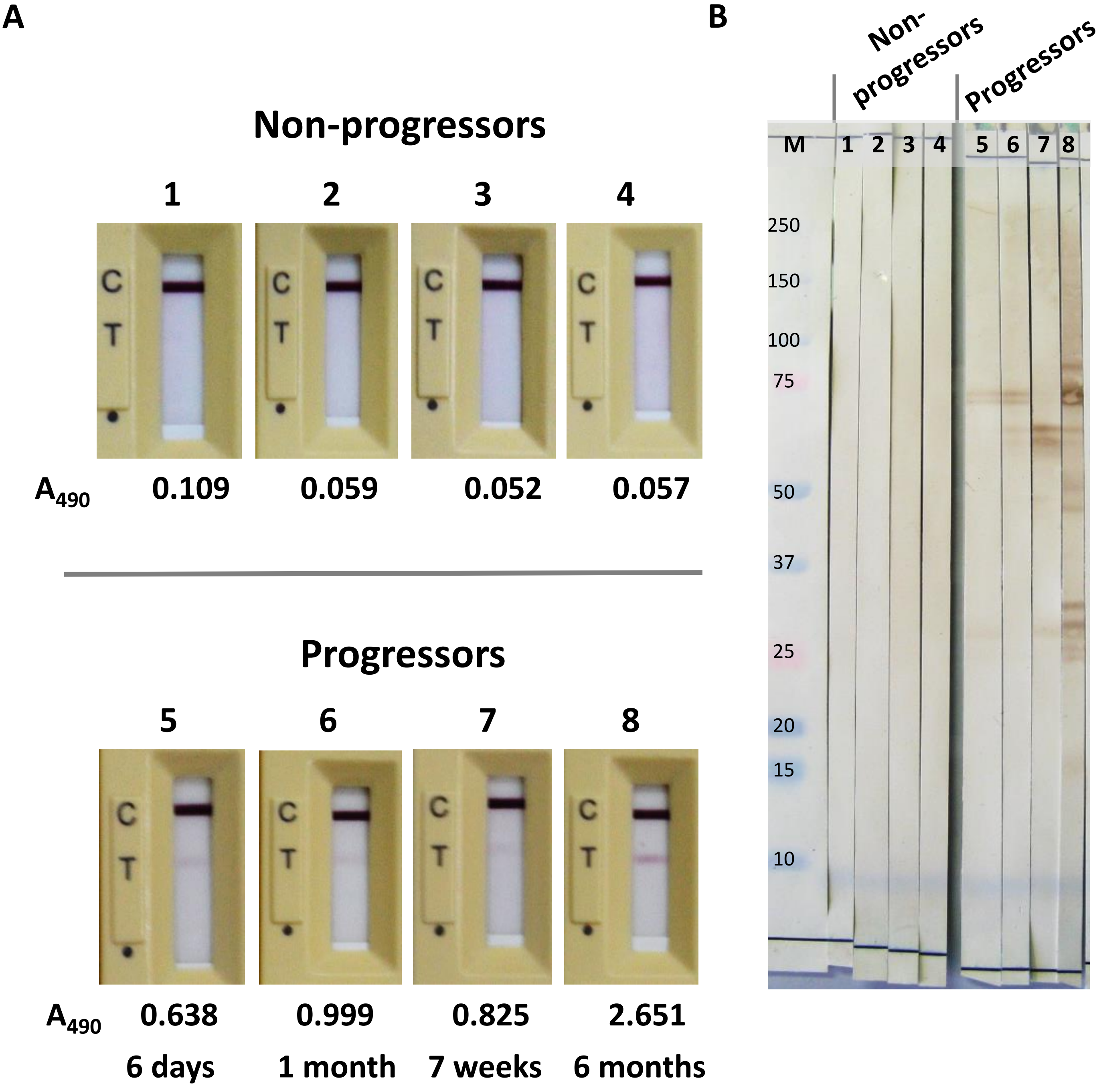

Figure S7. Elevated IgG1 in asymptomatic individuals was predictive of progression to symptomatic VL. 1-4, non-progressors: 5-8, progressors. (A) VL Sero K-SeT RDTs with corresponding IgG1 ELISA A_{490} readings below each RDT (ELISA cutoff = 0.128). C: control line; T: Test line. Time until active VL after sampling is given below the progressors, (B) western blots detecting IgG1 anti *L. donovani* for the same individuals. M: Molecular weight marker with kDa marked.
